# Curiosities of Bread

**Published:** 1862-10

**Authors:** 


// HALL'S JOURNAL OF HEALTH.

Our Legitimate Scope is almost boundless: for whatever begets pleasurable and harmless feelings, promotes Health; and whatever induces disagreeable sensations, engenders Disease.

WE AIM TO SHOW HOW DISEASE MAY BE AVOIDED, AND THAT IT IS BEST, WHEN SICKNESS COMES, TO TAKE NO MEDICINE WITHOUT CONSULTING A PHYSICIAN.

Vol. IX.] OCTOBER, 1862. [No. 10.

### CURIOSITIES OF BREAD.

THERE is Divine authority for saying that "bread is the staff of life." As to food, it is our mainstay; we never get tired of it; it is always palatable when we are hungry, as is cold water when we are thirsty. But cold water is made more refreshing, and bread is made more nutritious, by the introduction of a gas which, if breathed into the lungs in its unmixed, pure state, causes instant death!

We turn with disgust from eating any thing that is rotten, that is, in a state of decay; and yet, in the middle of the nineteenth century, the "loaf" upon our tables is itself rotten in part; the product of rottenness and whisky! But a new light has risen upon our world, for bread is now made which contains no alcohol, which does not require any putrefactive process, contains no alum, nor soda, nor saleratus; no emanation from the sweaty hands of a greasy cook, nor aught of those ten semi-circular lines of black which bound the digital extremities of the queen of the kitchen; nor does it ever become sodden or sour. This new bread is made of flour and water, into which, when mixed, is forced carbonic-acid gas; which, although so deadly to the lungs, makes all the difference between the sparkling water of the spring and the flatness of long-standing water, or that which has been once warmed; furthermore, from the time the flour is taken from the mill until the loaf is baked, human hands do not touch it.

Now, however much the mouths of our country friends may water at the very thought of so deliciously pure and clean an article, they must prepare themselves for some disappointment at the announcement, that it *is said* not to be economically made, except in large quantities. If every family made its own bread in this way, the great big bakery, like the Unitarian free-love home which flourished like Jonah's gourd on the opposite side of the way, would collapse immediately, if not sooner.

But how has "light bread" been made hitherto? By the aid of this same carbonic-acid gas, and in this wise: If water is mixed with flour simply, and the dough is thus baked, it is as heavy as lead, and as hard as a rock; because the flour is so fine, the particles lie close together, and form a compact, sodden mass; hence, it became necessary, in order to have the bread "light," to introduce something between the particles of flour, so as to keep them apart, and allow the heat to get around them and "cook" them. To accomplish this, an agency was necessary, as subtle and unsubstantial as thin air itself; otherwise, the heat would be kept away from each particle of flour, and would as effectually prevent the process of cooking as would the flour itself. To this end, some ancient gourmand set his wits to work, or else by some fortunate accident made the discovery, that "rising," or "yeast," introduced into the dough, and allowed to remain for a few hours, accomplished the object. Whether he got out a patent forthwith, or more generously spread his knowledge on the wings of the wind—as we doctors do, as soon as we are satisfied beyond all mistake or cavil that some valuable new remedy has been discovered—can not now be known; at all events, the patent has long since run out, and yeast and rising and leaven are all public property.

Fermentation and rottening are the vulgar and select names for one and the same thing, meaning "destructive decay," decomposition. When a thing is fermenting, bubbles are seen forming and rising and breaking; each bubble contains a light, thin air, called carbonic-acid gas; this gas, when a little warmed, begins to rise up through the dough, and would go up to the sky instanter, but it can't get out; it is a regular prisoner of war; it is literally bagged, surrounded, sewed-up, cabined, cribbed, confined; as helpless as a baby until it gets big, then it breaks away in high dudgeon, *nolens volens*, and scampers off to the regions of space. In the mean time, however, the bread has baked, and there is no further use for the prisoner at the bar; in fact, the more speedily he makes off with himself the better; for only until he has teetotally vamosed the ranche does the bread become "stale," and really fit to eat, healthfully speaking. Hence the propriety of exposing a new-baked loaf to the air.

But what prevents the carbonic-acid gas from escaping the instant it is formed? Flour contains a kind of glue; the gas rising, is caught by this glue, in the manner of pushing your finger upward under a spread newspaper, or the blowing up of soap-bubbles; each particle of gas expands as it gets warmer, and tends to carry away the detaining particle or sheet of gluten before it; and thus is made the numerous honey-comb cells which are seen on a cut loaf of bread. The eye can even discover, on the side of a large cell, a glazy or shiny lining; this is the dried gluten, bladder-like.

If the heat is too great, the carbonic-acid gas expands too rapidly, and bursts its envelope, as soap-bubbles will burst, if you blow too hard, and the bread will be heavy. If there is not warmth enough, the dough begins to decompose, to rot itself, and the bread is sour. But in the new process, the gas is forced in at once; and from the time the dough is mixed, until the bread is delivered from the oven, one hour passes. Hence, as no sour rising or yeast is put in the dough, there is nothing to communicate its sourness, and no time is allowed for fermentation to be originated. This is the only known method of producing absolutely pure wheaten bread of nature's own constituents; and doubtless the time will come when means will be devised for making "aërated bread" economically in families.

---

A Great Dietetic Truth.—The distinction between natural and artificial aliments, tonics, invigorators, etc., is this: the same amount of vigor or refreshment is uniformly imparted by the natural; half a pint of water will as effectually satisfy thirst to-day as it did twenty years ago or will twenty years to come, and so will a pound of bread as to hunger; but the unnatural, the artificial, as opiates, drugs, liquors, tobacco, etc., require to have constantly increasing quantities and in diminished intervals to have a given effect.

☞Next page (228) is continued from page 200.

Grey did fly around, for he knew by Johnson's manner all he bought that day would be cash to him. Tea, sugar, coffee, and soda were weighed out and tied up with almost lightning rapidity. A sack of flour was dragged from the back-room, and a cake of tallow and a pail of lard. Then calico was measured off, and muslin, and three pairs of little shoes done up, and then he paused a moment and said: "Any thing more?"

"Reckon up these, first."

"Nineteen dollars and fifty cents."

"Well, I guess I'll take the half-dollar in salt, pepper, cassia and nutmegs, and make it even. There," laying down four fives, "there goes my last dollar; but never mind, I'm square with the world now, and got something in the house besides. Could I borrow your wheelbarrow to trundle these things home?"

"Seventy dollars! what a godsend!" and the merchant added them to the four hundred. "If I could only raise thirty more—ah! Mrs. Benton," bowing politely to the minister's wife; "what can I do for you, to-day?" His heart lightened as he heard her ask for a pound of his best tea. That would put one dollar in his till, for he knew she never bought on credit. "A dollar's worth of sugar, fifty cents' worth of candles, four pounds of butter, eight pounds of lard—that's four dollars," he thought, as he weighed them. "Any thing more?"

"At the other counter, if you please."

Two pairs of children's shoes, calico for two dresses, muslin, linen and buttons for two shirts, come to six dollars—ten in all."

"Mr. Benton will call for the groceries; I'll only take these now," and handing him two fives, she started to go.

"Eighty dollars to-day—I must be in luck surely," and the merchant hastened to add the bills to the roll in his desk.

"Only twenty more, and I could weather the storm. Ah! Mrs. Nelson," bowing to the doctor's wife, "any thing in my line to-day? Shirting muslin? yes, I've some; some very nice," and he threw down half a dozen pieces.

"A whole piece, that's four dollars," he thought, as he laid it aside. "Children's shoes?"

"Yes, ma'am; some that I can warrant, too. Both pairs," and he tossed them into the piece of muslin. "Something for little boy's pants." "How'll this do? four yards, did you say? Something for jackets—here's the very thing—three yards—I'll throw you in the buttons and thread. Any thing else—ten dollars and a quarter—call it ten to-day, as it's cash down," and he bowed her out, and with a heart almost like a feather, placed the two fives in his desk.

"Ten more, and I'm saved. Ah! Mrs. Glenn, good evening; what can I show you? Cloth for pants—got the very thing, if it's for your husband; no shoddy about that," and he took it to the window that she might examine it the more closely. "I'll warrant it—three yards, did you say? I'll throw in lining, too," as he counted out buttons and flung down the thread; "I don't often do it these hard times. Red flannel, all wool; yes, some very soft, too; just the thing for weakly folks. I'll throw in silk to make it," seeing her hesitate a little. "There, just pick out all you think it'll take—don't leave me much profit, but—nine seventy-five—that pair of baby's shoes forty cents, but I'll call it twenty-five, and make it even change."

"Saved—saved at the last moment," and he hastened to write his letter, and then depositing the bills in it, he carried it to the office, running every step, lest the mail should be made up. An hour later, and he was singing to little Charlie as he hadn't sung for a fortnight, and jumping the little fellow till the young mother declared every bone in the child's body would be broken. "I'll risk him," said the happy father, tossing him again quite to the ceiling; "I'll risk any thing to-night. Wasn't it a providence, though, Nelly, that at the very last hour a hundred dollars should have been paid in? How on earth so much money got into the village, just then, is a mystery to me. But for *it*, where should we be now?"

Backward and forward went the Boston merchant; his steps were quick and nervous, and then slow and irresolute. Every few minutes he glanced at the clock. How swift went the hands. Now it strikes two. Another hour and his fair name, his name, that for twenty years has stood unblemished, would be bandied about in the mouths of vulgar men. "And all for a paltry five hundred dollars, a sum that in ordinary times I carried daily about me. What did he say?" to a clerk who entered in breathless haste. "'Nothing over'—there goes my last hope, for that Grey will pay up, is out of the question. If we city merchants are so crowded, what a strait those poor country fellows must be in!"

Backward and forward he walked again—the clock struck the quarter. "Mail is distributed by this time. Run, John, to the office, and if there is a letter, fly with it. Pshaw! why did I send him? It's shoe-leather worn for nothing. Five minutes—ten minutes—Heavens, why don't he come? it may as well be over now—a letter, pshaw—quick," and he tore it open. Without stopping to read it, he grasped the bills and counted. Then seizing a roll from his desk, and jerking his hat from its hook, he ran, yes, fairly ran to the bank. The five thousand dollars were counted out by the teller, his note was given up, and he was half-way home when the clock struck three.

"I couldn't wait till dinner-time, Lizzie, I couldn't wait," he exclaimed, clasping a beautiful woman in his arms. "I've run every step of the way to tell you the news. We're saved, we're saved."

Two firms saved from failure—four families rescued from want, and how many, many hearts made light and glad by the self-sacrifice of one farmer's wife! Oh! this "making do" is verily, we believe, the best and cheapest remedy for these hard times that crush us all so terribly. Suppose we try it, not one, but all of us. It is said that a pebble thrown into the ocean causes a vibration that does not cease till the opposite shore is touched. How far may not one dollar, saved from family expenses, and turned towards the payment of our honest debts, how far may not *it* go towards bringing back to our loved and beautiful land the good times for which we are all sighing! Why not make pebbles of our dollars, and cast them hopefully into the great ocean of want?

